# Replication of Known and Identification of Novel Associations in Biobank-Scale Datasets: A Survey Using UK Biobank and FinnGen

**DOI:** 10.3390/genes15070931

**Published:** 2024-07-17

**Authors:** Alexander A. Tkachenko, Anton I. Changalidis, Evgeniia M. Maksiutenko, Yulia A. Nasykhova, Yury A. Barbitoff, Andrey S. Glotov

**Affiliations:** Department of Genomic Medicine, D.O. Ott Research Institute of Obstetrics, Gynaecology, and Reproductology, 199034 St. Petersburg, Russia; castorfiber@list.ru (A.A.T.); anton@bioinf.me (A.I.C.); jmrose@yandex.ru (E.M.M.); yulnasa@gmail.com (Y.A.N.); anglotov@mail.ru (A.S.G.)

**Keywords:** GWAS, genome-wide association, replication, SNP, meta-analysis

## Abstract

Over the last two decades, numerous genome-wide association studies (GWAS) have been performed to unveil the genetic architecture of human complex traits. Despite multiple efforts aimed at the trans-biobank integration of GWAS results, no systematic analysis of the variant-level properties affecting the replication of known associations (or identifying novel ones) in genome-wide meta-analysis has yet been performed using biobank-scale data. To address this issue, we performed a systematic comparison of GWAS summary statistics for 679 complex traits in the UK Biobank (UKB) and FinnGen (FG) cohorts. We identified 37,148 index variants with genome-wide associations with at least one trait in either cohort or in the meta-analysis, only 3528 (9.5%) of which were shared between UKB and FG. Nearly twice as many variants (6577) were replicated in another dataset at the significance level adjusted for the number of variants selected for replication. However, as many as 9230 loci failed to be replicated. Moreover, as many as 5813 loci were observed as significant associations only in meta-analysis results, highlighting the importance of trans-biobank meta-analysis efforts. We showed that variants that failed to replicate in UKB or FG tend to correspond to rare, less pleiotropic variants with lower effect sizes and lower LD score values. Genome-wide associations specific to meta-analysis were also enriched in low-effect variants; however, such variants tended to be more common and have more consistent frequencies between populations. Taken together, our results show a relatively high rate of non-replication of genome-wide associations in the studied cohorts and highlight both widely appreciated and less acknowledged properties of the associations affecting their identification and replication.

## 1. Introduction

Over the last decades, numerous genome-wide association studies (GWAS) have been performed to identify the risk loci for human complex traits and diseases [1,2]. GWAS has been remarkably successful in pinpointing key susceptibility loci for isolated cohorts, and the rate of results replicability for GWAS findings is greater compared to candidate gene association studies. For example, the majority of new genome-wide associations reported in the GWAS Catalog [3] correspond to the replication of previously reported associations [4]. At the same time, only 40% of previously reported genome-wide associations are rediscovered at genome-wide significance level. Understanding of the determinants of the replicability of GWAS results may be helpful both for the general understanding of complex trait biology and for the design of future GWAS studies, including large-scale trans-ethnic efforts.

Rapid development of biobanking and the establishment of large-scale projects such as the UK Biobank [5] or FinnGen [6] have pushed GWAS beyond single cohorts and individual traits, allowing for complex multivariate analyses and trans-biobank meta-analyses [7]. While trans-biobank association studies are usually performed for single traits or groups of related traits, several recent studies have demonstrated how such an approach can be broadened by comparing genome-wide associations for hundreds of complex traits between different biobanks. A notable example comes from the work of Sakaue et al. [8], who have performed a meta-analysis of GWAS results from the UK Biobank and Biobank Japan (BBJ) for 220 human complex traits. The meta-analysis revealed 8653 novel associations for biomarker, disease, and medication usage endpoints even with the strictest significance cut-offs. A matrix decomposition of GWAS summary statistics for Japanese and European individuals allowed the identification of latent genetic components that explain diseases of similar etiologies. Sakaue et al. [8] also demonstrated the reproducible properties of pleiotropic loci, such as their enrichment with recent positive selection signals.

A peculiar benefit from the large-scale comparison and meta-analysis of GWAS results for hundreds of complex traits is the ability to identify important properties of these studies and/or genetic variants that affect the reproducibility and reliability of the identified associations. In our work, we made an effort to systematically compare the summary statistics of GWAS from more than six hundred pairs of complex traits in the UK Biobank and FinnGen projects, focusing on the properties of genetic variants that correlate with the rate of replication. We demonstrated that the replication of genome-wide associations in biobanks is affected not only by well-established factors, such as effect sizes and minor allele frequencies, but also by the variant’s LD score and the degree of pleiotropy. Finally, we described the key properties of genetic variants with unique associations in cross-cohort meta-analysis.

## 2. Materials and Methods

### 2.1. Data Collection

We used publicly available meta-analysis results from the FinnGen project website (https://public-metaresults-fg-ukbb.finngen.fi/about, accessed on 18 November 2023). The dataset included 679 phenotype pairs and was created using the European subset of pan UKBB study and FinnGen data from freeze 9. Full summary statistics were downloaded from google cloud bucket gs://finngen-public-data-r9/ukbb_meta/ (accessed on 25 November 2023). All listed genome positions in the dataset are on the GRCh38 human genome version. The initial FG dataset comprises 377,277 individuals (210,870 females and 166,407 males). A total of 20,175,454 variants were analyzed for 2272 phenotypes, of which 679 were included in the meta-analysis. The initial UKB dataset was obtained for 420,531 individuals (mostly of European ancestry) and included 7200 endpoints. A total of 28,987,534 variants were characterized. Some endpoints were modified by the authors of meta-analysis to better suit the FG definition of phenotypes. The overall number of variants for meta-analysis after combining FG and UKB datasets amounted to 31,654,403. Out of them, only 12,265,962 SNPs overlapped between biobanks, and they were used for subsequent analyses. Numbers of cases and controls for particular analyzed phenotypes are listed in Supplementary Table S1, which includes a publicly available manifest of the meta-analysis from https://public-metaresults-fg-ukbb.finngen.fi/about, (accessed on 25 November 2023).

### 2.2. Identification and Comparison of Associated Variants and Loci

First, we calculated the total number of SNPs associated with any given trait in either UKB or FG at a genome-wide significance level of *p* < 0.05/number of analyzed variants (12,265,962). 

To identify the independent genomic regions associated with each trait, we used the linkage disequilibrium (LD)-based clumping function in PLINK v1.90b6.21 [9]. Default PLINK settings (physical distance cutoff of 250 kb, r^2^ > 0.5) and a *p*-value cutoff of *p* < 0.05/number of analyzed variants) were used to generate clumps of variants. To assess the overlap of risk loci for the same trait in different biobanks, clumps generated by PLINK were transformed into 100 kb-wide intervals around the index variant coordinates, sorted, and merged using BEDtools v. 2.26 [10]. Merged intervals obtained using UKB, FG, or UKB+FG meta-analysis data were then intersected for each trait, in either a pairwise manner or for all three sources simultaneously. Additionally, correspondence between risk loci was ascertained based on the set of index SNPs in each source of summary data.

Index variants from clumps (=lead variants) were additionally grouped by LD estimates based on European subset of 1000 Genomes project data using PLINK v1.90b6.21. Variants in a 1 Mb window and less than 100 SNPs apart from each other were considered to be in LD if the *r^2^* estimate exceeded the cut-off of 0.5.

All data were gathered into two summary tables indexed by trait pairs (the table contained summary association data for each pair of traits across all sources) or by variant (the table contained summary association data for each variant across all sources). Variants were additionally annotated with their LD scores according to the data of Broad Institute [11] (accessed on 8 February 2023), as well as the scaled difference in MAF between data sources. The latter value was calculated as the absolute value of difference between MAF in FG data and UKB data divided by the maximum among MAF values from FG and UKB for this pair of traits.

### 2.3. Definition of Replicated and Non-Replicated Associations

For the purpose of this study, the following algorithm was employed for identification of reproducible associations: (1) a set of all index variants was selected from either UKB or FG; (2) a target significance threshold *α* was calculated as 0.05/*n*, where *n* is the number of index variants selected in step (1); and (3) the association of a given variant with same trait(s) in another biobank was checked, requiring *p* < *α*. If a variant was associated with several traits, at least one overlapping trait was required to consider an association of a variant as replicated.

Additionally, we assessed the expected degree of replication between FG and UKB based on the method in Okbay et al., 2016 [12] (the method is outlined in Supplementary Material 1.8.3). Lead variants after clumping with PLINK (described above) were used to calculate probabilities that their estimates in respective biobanks were significant at a significance level of *p* < 0.05/number of analyzed variants (12,265,962). Summation of these probabilities was used to calculate the expected amount of replicating variants.

Meta-analysis specific associations (“meta-specific variants”) were defined as variants that were marked as index ones in the UKB+FG meta-analysis but did not reach a genome-wide significance threshold in either of the source datasets.

## 3. Results

### 3.1. Characterization of Genome-Wide Significant Loci in UK Biobank and FinnGen

UK biobank (UKB) [5] and FinnGen (FG) [6] are the two largest publicly available resources that provide GWAS summary statistics for thousands of traits in individuals of European ancestry. The large number of traits (endpoints) for which GWAS summary statistics are available from both cohorts, as well as the peculiar genetic history of the Finnish population, make these two datasets a perfect resource to systematically investigate the replication of genome-wide associations in genetically distinct populations.

We used the available mapping of FG and UKB phenotypes from panUKBB-FinnGen meta-analysis. Matching includes some custom UKB phenotypes and, overall, contains 679 phenotype pairs. In total, we discovered 8207 distinct lead SNPs associated with at least one of the 679 traits in UKB, and 19,514 in FG.

**Figure 1:**
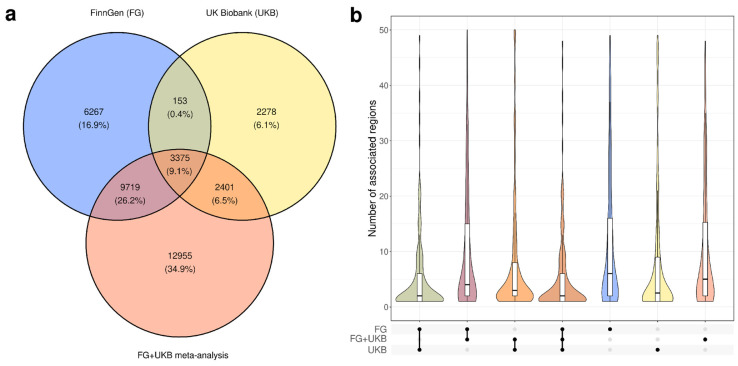
General overlap between significant genome-wide associations observed in UK Biobank (UKB), FinnGen (FG), and UKB+FG meta-analysis. (**a**) Venn diagram showing the overlap between sets of lead SNPs at genome-wide significant loci based on FG, UKB, and meta-analysis data. (**b**) Plots showing the number of associated genomic regions (100,000 base pairs around each lead SNP) detected for each trait in UKB, FG, and UKB+FG meta-analysis separately, as well as shared by indicated combinations of datasets. The colors of the violins match the colors of the corresponding sectors of the Venn diagram on (**a**).

To assess the replication of genome-wide associations between UKB and FG, we also analyzed the genome-wide meta-analysis results publicly available from FG. A total of 28,450 lead SNPs were identified at genome-wide significant loci in the meta-analysis results, which is greater than the combined number of index variants identified in UKB and FG. We next analyzed the overlap in the sets of lead SNPs at genome-wide significant loci associated with at least one trait in UKB, FG, or the UKB+FG meta-analysis (Figure 1). We found that only 3375 loci (9.1%) were discovered in all three datasets. The lowest amount of shared lead SNPs was observed for the FG/UKB comparison, with as much as 15,986 (81.9%) FG associations not observed in UKB, and 4679 (57.0%) UKB associations not observed in FG (Figure 1a). The overlap was much larger when FG or UKB loci were compared to the UKB+FG meta-analysis results, with 67.1% (13,094 of 19,514 index variants) of FG loci and 70.4% (5776 of 8207 index variants) of UKB loci found as index variants in the meta-analysis results (Figure 1a). These results emphasize that the replication of associations between cohorts at genome-wide significance level is much less probable than the replication using genome-wide meta-analysis.

Notably, as many as 12,955 lead SNPs were found exclusively in the meta-analysis (Figure 1a, bottom section). Furthermore, we found 153 lead variants that were observed in both UKB and FG, but not in the meta-analysis. All of these 153 variants were associated with distinct traits in UKB and FG and were not replicated in any trait-wise meta-analysis.

The relatively low level of overlap between sets of lead SNPs at genome-wide significant loci between the two datasets may be, at least in part, explained by different index variants tagging the same locus in different populations. Hence, we next went on to analyze the overlap between the genomic regions bearing significant genome-wide associations in the two datasets. Analysis of the overlap between all genome-wide significant loci (defined as clump intervals generated by PLINK) confirmed that more non-overlapping loci are observed in FG. A vast majority of these loci are not directly replicated in the UKB, and genome-wide significant associations are observed in all three sources for only 21.7% of non-overlapping regions (Supplementary Figure S1). However, to avoid overlaps between regions associated with different traits at the same chromosomal locus as well as marginally overlapping regions, we next went on to consider the overlap of loci for individual trait pairs.

Similarly to the results obtained using sets of lead SNPs, we observed that FG had, on average, more associated loci than UKB (Figure 1b), with the corresponding average number of loci associated with a single trait in the UKB+FG meta-analysis falling in between them. The higher number of associations in FG is probably due to the higher number of cases for corresponding phenotypes in FG (Supplementary Figure S2) and, consequently, the higher power of association analysis even though UKB has a larger overall cohort size (420,531 vs. 377,277 in FinnGen). At the same time, fewer than 10 loci were detected for the majority of traits in all of the three data sources. According to the results shown in Figure 1b, the average number of overlapping regions bearing genome-wide associations in both UKB and FG was much lower compared to either dataset (Figure 1b). In the vast majority of cases, more loci were shared between UKB or FG and meta-analysis results than between the two source datasets.

We also investigated the correlation regarding the per-trait number of associated loci between UKB, FG, and meta-analysis. We found that, while weak correlation was detected in all three pairwise comparisons (Supplementary Figure S3), a substantial difference in the number of risk loci identified in UKB and FG could be seen in many cases. The results suggest that, while FG boasts greater power of association analysis (Figure 1), the UKB dataset may provide more insights into the genetic architecture of particular traits. This assumption is corroborated by the analysis of the number of cases for each trait (Supplementary Figure S1), which yields results similar to those of Kurki et al. [6].

### 3.2. Analysis of the Factors Affecting Replication of Genome-Wide Associations

The discordance between genome-wide associations identified using the UKB or FG cohort may be, at least in part, explained by differences in the study design, data collection and analysis strategies. At the same time, the analyzed dataset allows us to investigate the variant-level determinants that contribute to the replication (or non-replication) of the detected genome-wide associations.

To take a closer look at such determinants, we created a summary table containing all associations in UKB, FG, and UKB+FG meta-analysis and the level of individual SNPs. The UKB and FG datasets contain a total of 31,654,403 different SNPs and 12,265,962 of them overlap between biobanks (see Methods for details). Of these, 301,375 (~2.46%) were found to be associated with at least one phenotype pair from FG, UKB, or UKB+FG meta-analysis, and 37,148 were index variants in at least one data source. Among 37,148 index variants, there were 18,563 variants that could be combined into 4750 groups of variants in LD (see Methods) containing at least two variants each. Additionally, 805 of these groups contain linked variants that have been observed as index variants in different biobanks.

For the analysis of the variant-level determinants of replicability, we focused on the 23,335 index variants remaining after selecting only one representative SNP from each group of SNPs in LD. A variant was deemed reproducible if it was an index variant in one dataset (FG or UKB) and passed the *p*-value threshold of (0.05/number of variants selected for replication, see Methods) in another biobank and the direction of the effect was consistent (a total of 43 variants had discordant direction of effects). Otherwise, variants were considered non-replicating. The two resulting groups of index variants included 6577 (28.2%) and 9230 (39.5%) SNPs, respectively. Note that the percentages do not add up to one hundred percent because we purposefully excluded a group of meta-analysis-specific associations (5813, 24.9%) that will be discussed later, as well as variants reaching genome-wide significance that were marked as index ones in meta-analysis. Importantly, the number of successfully replicating variants identified was lower than the number predicted using effect size estimates (6718 for FG and 5500 for UKB) (see Methods Section 2.3). This finding, however, may be (at least, in part) explained by the winner’s curse, which has not been adjusted for in our analysis.

**Figure 2:**
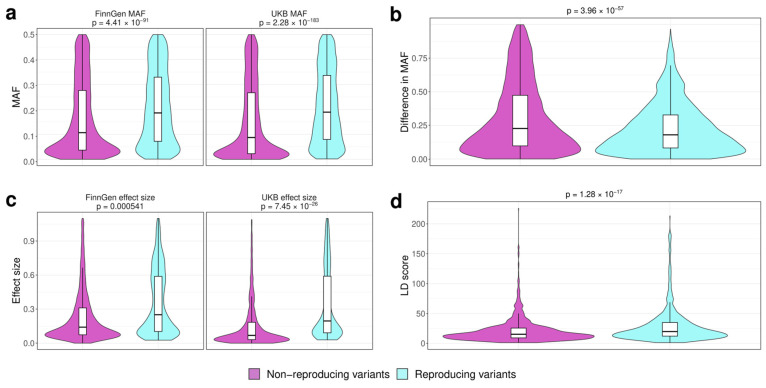
Comparison of variant-level properties between index SNPs at loci with reproducible (blue) and non-reproducible (purple) associations. Plots show (**a**) minor allele frequency (MAF) (left—FinnGen (FG), right—UK Biobank (UKB)); (**b**) scaled difference in MAF between UKB and FG; (**c**) variant effect sizes (left—FG, right—UKB); and (**d**) variant LD score [11].

Having split the variants into these groups, we next went on to compare different properties of variants between the groups. The minor allele frequency (MAF) is one of the most important and well-studied parameters that affects the power of association analysis. Indeed, we observed that replicating variants had a higher median MAF both for UKB (0.189 vs. 0.0875 for non-replicating ones) and for FG (median MAF 0.186 vs. 0.107) (Wilcoxon test *p*-values 2.28 × 10^−183^ and 4.41 × 10^−91^ for FinnGen and UKB, respectively, Figure 2a). We next questioned if the differences in MAF between populations contribute to the rate of replication, as suggested by Kurki et al. [6]. To test this, we computed the scaled difference in MAF values between the studies (see Methods) and compared this value between the two variant groups. We found significant differences in scaled MAF differences (Wilcoxon test *p*-value = 3.96 × 10^−57^) (Figure 2b); also, non-replicating variants were significantly enriched with those that had a high (>0.5) scaled MAF difference (2156/9230 vs. 680/6577 for replicating variants, chi-squared test *p*-value < 2.2 × 10^−16^).

While more common variants have a greater chance of being replicated due to statistical reasons, such variants also usually have a lesser effect on the phenotype. Hence, we next compared the effect sizes for the replicating and non-replicating variants in both the UKB and FG cohorts. We found that, despite their higher average MAF, replicating genome-wide associations correspond to loci with a higher effect on the phenotype (Wilcoxon test *p*-value = 5.41 × 10^−4^ and 7.45 × 10^−26^ for FG- and UKB-based effect size estimates, Figure 2c). Finally, the failure of the association to be replicated may be caused by the fact that the observed lead SNP is not the actual causal one but is, rather, in high LD with it. If this assumption is true, replicating variants could be expected to have a higher LD score, indicating their linkage to more variants in the locus. We evaluated this hypothesis by comparing the LD score values for SNPs in the two groups. In accordance with our assumption, replicating variants showed a slightly greater median LD score value (Wilcoxon test *p*-value = 1.28 × 10^−17^) (Figure 2d).

Having characterized the major properties of replicating associations, we then questioned if the observed differences depend on the source of the variants used in the replication. To answer this question, we compared all of the aforementioned variant properties for replicating and non-replicating associations derived from FG (4074/7960) and UKB (2966/1403) separately. In both cases, the differences in MAF, effect sizes, and LD scores were concordant with the combined analysis (Supplementary Figures S4 and S5), indicating that the observed determinants of association replicability are independent of the initial signal source.

**Figure 3:**
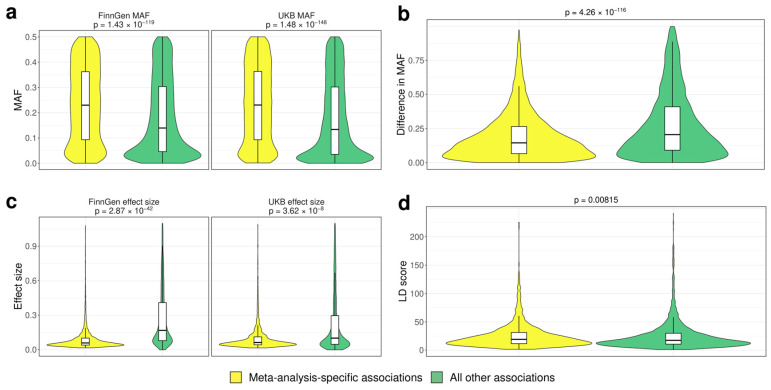
Comparison of variant-level properties between index SNPs at loci showing exclusive genome-wide association in trans-biobank meta-analysis (yellow) and all other loci (green). Plots show (**a**) minor allele frequency (MAF) (left—FinnGen (FG), right—UK Biobank (UKB)); (**b**) scaled difference in MAF between UKB and FG; (**c**) variant effect sizes (left—FG, right—UKB); and (**d**) variant LD score [11].

We next turned our attention to a specific group of SNPs that were associated with phenotypes on the genome-wide significance level only in the meta-analysis results (here and later, such variants are termed “meta-specific” SNPs). Overall, we found 5813 such meta-specific SNPs associated with 200 distinct phenotype pairs. We decided to compare the properties of such variants to other ones with at least one genome-wide association in our dataset. Such a comparison revealed that meta-specific SNPs have significantly higher MAF in both the FG (Wilcoxon test *p*-value = 1.43 × 10^−119^) and UKB (Wilcoxon test *p*-value = 1.48 × 10^−148^) datasets (Figure 3a). Rare (MAF < 0.1) ones were underrepresented among the meta-specific variants, while the prevalence of common (MAF close to 0.5) variants in this group was increased. The scaled difference in MAF between UKB and FG was also significantly (Wilcoxon test *p*-value = 4.26 × 10^−116^) lower for meta-specific SNPs (Figure 3b). This might mean that meta-specific variants are more likely to be picked up during meta-analysis due to similar effect sizes and MAFs in the two cohorts. Meta-specific SNPs also had lower degrees of pleiotropy (Supplementary Figure S6). In contrast to variants that replicate their association at the genome-wide significance level, meta-specific variants, despite their larger MAF, have smaller effect sizes in both FG and UKB data (Wilcoxon test *p*-value 2.87 × 10^−42^ and 3.62 × 10^−8^, respectively, Figure 3c). This finding could explain their failure to reach genome-wide significance level in the initial analysis. Finally, we compared the LD scores for meta-specific and non-meta-specific variants and found that LD scores were slightly lower for meta-specific variants (Wilcoxon test *p*-value = 0.00815) (Figure 3d).

## 4. Discussion

GWAS results have proven to be highly reproducible, especially if the researchers adhere to the main principles of validating and reporting their findings [4]. At the same time, the rapid development of biobanks and the creation of numerous large-scale genotypic and phenotypic datasets for different ancestries necessitates an unbiased analysis of the consistency of GWAS results in biobank-scale cohorts, as well as of genetic factors that influence the replicability of genome-wide associations and the ability to detect novel associations in the genome-wide meta-analysis. To this end, in our work, we performed a systematic comparison of GWAS results in UKB and FG cohorts.

The original FinnGen study reported that a significant proportion of novel associations from the FG cohort correspond to the variants that are enriched in the Finnish population compared to other Europeans [6]. Notably, our results (which are based on a larger number of trait pairs and include pairwise genome-wide meta-analysis) confirm a substantial role of allele frequency differences in the replicability of genome-wide associations, either in the other cohort or in the genome-wide meta-analysis (Figure 2). Besides the technical reasons, the lower replication rates for the variants with lower effect sizes and larger between-population variance in frequency may suggest a role for positive selection. Indeed, certain genetic variants may be important for adaptation to the local environment, and recent positive selection can thus contribute to the differences in variant allele frequency between populations. However, due to a pronounced founder effect in the Finnish population, further studies in more diverse ethnic groups are needed to fully understand the nature of allele frequency differences for non-replicating genome-wide associations, if this difference persists across other datasets.

A question of population-specific rare variants highlights a certain set of the limitations of multiethnic population studies based on common variation like this one. Rare variants are widely accepted [13] as an important component of genetic architecture of human diseases, yet we are unable to properly take them into account. A similar issue is connected to runs of homozygosity that were shown to drive pathogenic variation [14] and epigenetic variation between populations [15]. Both of these factors might contribute to differences between populations in this study and should be thought of as parts of a bigger picture encompassing all contributions to complex trait development.

The inherent link between MAF and effect size of a variant has been thoroughly investigated since the advent of GWAS [16]. Indeed, our analysis confirms a widely known assertion that both MAF and effect size are good predictors of association replicability. It is important to note that the replicating variants tend to have a higher effect size in both cohorts despite having a larger MAF (Figure 2), and this pattern does not depend on the source of the initial association (Supplementary Figures S4 and S5). This observation may be explained by either (i) the greater degree of ancestry specificity for rare variants or (ii) the lower power to detect association for rare variants. Indeed, one might expect that rare variants will be observed in fewer individuals in a cohort, thus decreasing the statistical power to detect the association even if the true effect size is relatively large.

Yet another intriguing observation is the higher degree of pleiotropy for reproducible associations (Supplementary Figure S6). Pleiotropy is an important phenomenon that shapes the genetic architecture of all traits [17]. Pleiotropic effects in GWAS, however, are much more typical for common variants [18]. Hence, the observed enrichment of reproducible associations with pleiotropic variants may be driven not by functional or evolutionary factors, but rather by the greater statistical reliability of common variants (as discussed in the previous paragraphs). Another important issue is that we did not group traits by their genetic correlation, which may inflate the observed degree of pleiotropy for many variants [18].

An important issue in genome-wide association studies is the ability to identify a causal variant among those present at the associated locus. This task is usually solved using methods such as statistical and functional fine mapping [19]. When comparing the results of GWAS from different cohorts, one has to bear in mind that overlapping associated loci may actually tag different causal variants. To address this question, multiple statistical methods have been proposed to test for co-localization of GWAS signals at a particular locus (e.g., [20]). Our results suggest that the LD score of a variant is an important factor affecting the reproducibility of genome-wide associations (Figure 2). This finding may indicate that, indeed, different variants with a low MAF may be responsible for the association at the same locus in different populations.

Notably, 24.9% of the lead variants identified in our analysis represent unique associations observed in genome-wide meta-analysis. This finding emphasizes the importance of trans-biobank association studies. Among such meta-analysis-specific associations, arguably the most interesting are those that correspond to traits without a single genome-wide significant hit in either biobank. In total, 47 such novel associations were observed for as many as 24 traits (Supplementary Table S2). Table 1 illustrates some of the most notable examples. These include the association of rs3777781 (6:133247575:T:A) in *EYA4* with Ménière disease, a gene that has previously been suggested to have a role in hearing loss [21] due its widespread expression in cochlear ducts in mice. In another case, UKB+FG meta-analysis pinpoints the role of rs167479 variant in *RGL3* in pre-eclampsia. This finding corroborates our recent results showing the association of *RGL3* variants with general hypertensive disorders of pregnancy [22]. Finally, we observed the association of the rs73585022 variant in *OPCML* with sexual dysfunction. *OPCML* encodes an opioid receptor that is predominantly expressed in the brain; however, this gene has also been involved in tumorigenesis and has been found to be expressed in the reproductive tract in mice [23]. The gene has multiple known associations, according to the GWAS Catalog [3]; however, it has not been previously implicated in sexual behavior.

Taken together, our analysis demonstrates the utility of trans-biobank genome-wide meta-analysis for the identification of robust genetic markers of complex traits. It further highlights both well-established (such as larger MAF consistency and greater estimated effect size) and less acknowledged (larger LD score and degree of pleiotropy) factors that are positively correlated with replicability of genome-wide associations. In addition, we show that the novel loci identified using genome-wide meta-analysis of large-scale GWAS primarily comprise common, low-effect variants with consistent frequencies across populations. Thus, our analysis shows important properties of genetic variants that can be taken into account when conducting cross-cohort association studies and designing robust polygenic risk scores (e.g., by including a subset of variants with lower variation in MAF) for predicting human complex traits in different populations. 

## Figures and Tables

**Table 1:** Examples of genome-wide associations that are observed only in the FG+UKB meta-analysis results.

Trait	rsID	Gene	MAF (UKB/FG)	*β* (UKB/FG)	*p*-Value (UKB/FG)	Meta *p*-Value
Ménière disease ^1^	rs3777781	*EYA4*	0.22/0.30	−0.15/−0.17	1.57 × 10^−2^/2.89 × 10^−8^	1.46 × 10^−9^
Pre-eclampsia ^2^	rs167479	*RGL3*	0.47/0.42	0.06/0.10	1.61 × 10^−3^/1.63 × 10^−8^	1.28 × 10^−10^
Sexual dysfunction ^3^	rs73585022	*OPCML*	5.7 × 10^−3^/8.3 × 10^−4^	3.68/2.25	9.97 × 10^−8^/8.23 × 10^−5^	1.29 × 10^−10^

^1^—H8_MENIERE; ^2^—O15_PREECLAMPS; ^3^—F5_SEXDYS.

## Data Availability

Data and code pertinent to the analysis presented in this work are available at: https://github.com/mrbarbitoff/ukb_fg_comparison/.

## Supplementary Materials

The following supporting information can be downloaded at: https://www.mdpi.com/article/10.3390/genes15070931/s1, Figure S1: A Venn diagram showing the overlap between the independent loci bearing genome-wide significant associations in different datasets; Figure S2: A scatterplot showing the numbers of cases in different biobanks for the matching trait pairs used in our analysis; Figure S3: A hexagonal scatterplot showing the relationship between the number of associated genomic regions in the UKB, FG, and UKB+FG meta-analysis, for the matching trait pairs used in our analysis; Figure S4: Comparison of variant-level properties between FinnGen-sourced index SNPs at loci with reproducible (blue) and non-reproducible (purple) associations; Figure S5: Comparison of variant-level properties between UK Biobank-sourced index SNPs at loci with reproducible (blue) and non-reproducible (purple) associations; Figure S6: Barplots showing the proportion of pleiotropic and non-pleiotropic variants among different groups of variants. Table S1: Summary of the genome-wide associations for the 679 trait pairs. Table S2: Genome-wide associations specifically identified in the genome-wide meta-analysis of UKB and FG data for traits that do not have any significant hits in either of the source datasets.
