# Supplementary material for: Replication of Known and Identification of Novel Associations in Biobank-Scale Datasets: A Survey Using UK Biobank and FinnGen
Source: Genes (Basel). 2024 Jul 17;15(7):931. doi: 10.3390/genes15070931 (PMC11275374; doi:10.3390/genes15070931)
Supplement: Supplementary file 1 [file genes-15-00931-s001.zip › Supplementary Figures.pdf]

Systematic comparison of genetic effects across UK Biobank and FinnGen reveals key determinants of association replicability

Alexander A. Tkachenko , Anton I. Changalidis, Evgeniia M. Maksiutenko, Yulia A. Nasykhova, Andrey S. Glotov and Yury A. Barbitoff

Supplementary Figures

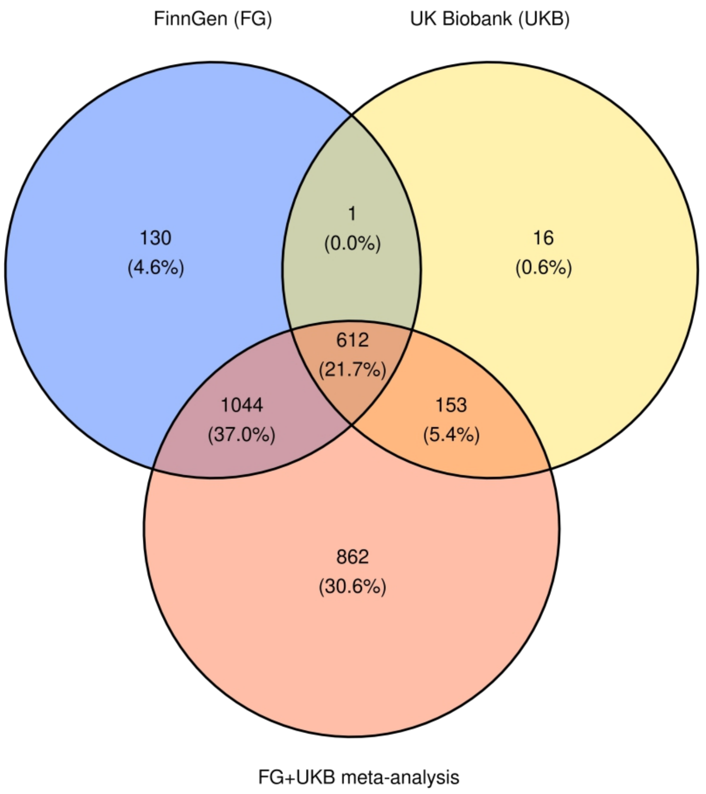

Supplementary figure S1. A Venn diagram showing the overlap between the independent loci bearing genome-wide significant associations in different datasets. The numbers were obtained by merging all genomic intervals of all loci identified across datasets using BEDtools `merge` function and calculating the proportion of loci in each section of the diagram.

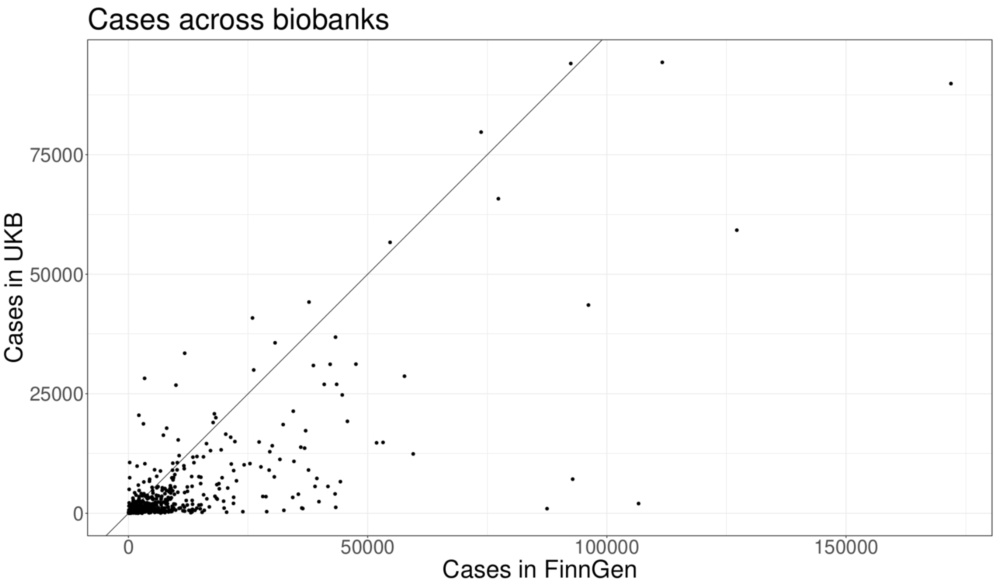

Supplementary figure S2. A scatterplot showing the numbers of cases in different biobanks for the matching trait pairs used in our analysis, X axis - FinnGen, Y axis - UKB

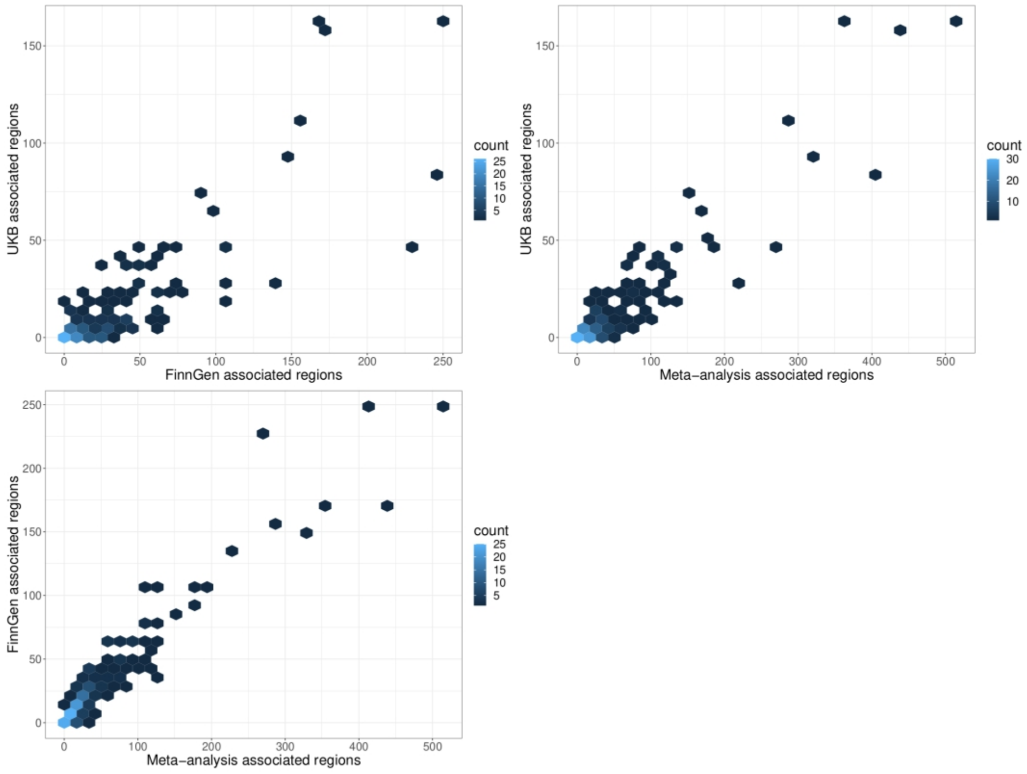

Supplementary figure S3. A hexagonal scatterplot showing the relationship between the number of associated genomic regions in the UKB, FG, and UKB + FG meta-analysis, for the matching trait pairs used in our analysis. Color of the hexagon is proportional to the number of traits with a given ratio of associations,

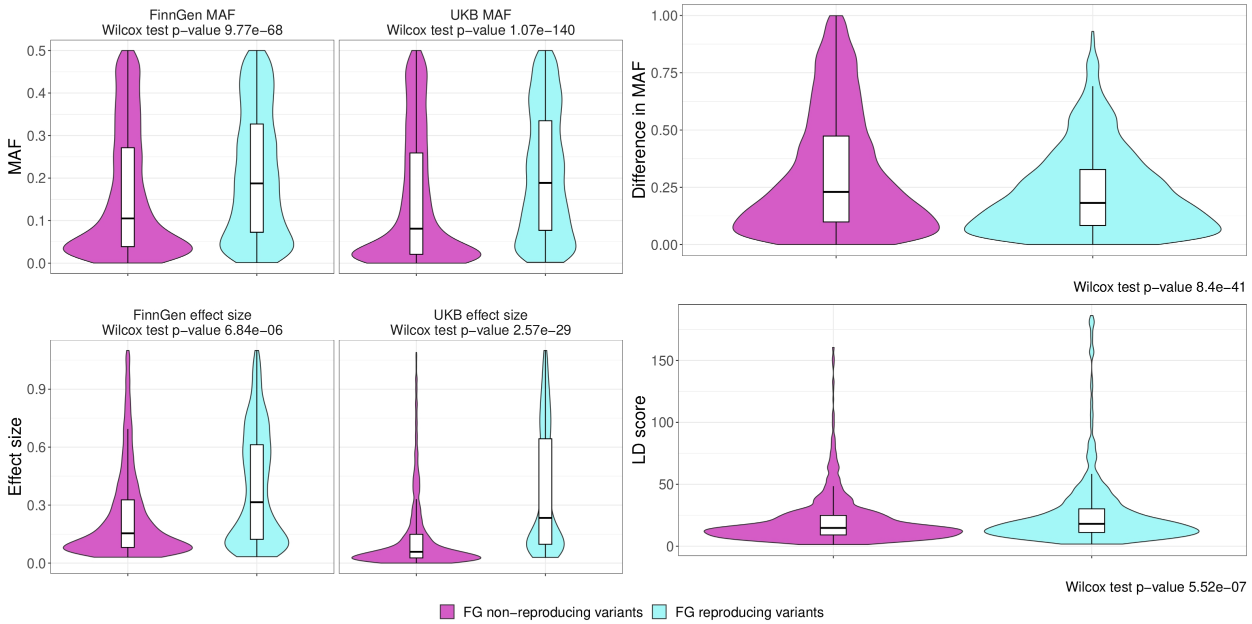

Supplementary figure S4. Comparison of variant-level properties between FinnGen-sourced index SNPs at loci with reproducible (blue) and non-reproducible (purple) associations. Plots show (a) minor allele frequency (MAF) (left - FinnGen (FG), right - UK Biobank (UKB)); (b) scaled difference in MAF between UKB and FG; (c) variant effect sizes (left - FG, right - UKB); and (d) variant LD score.

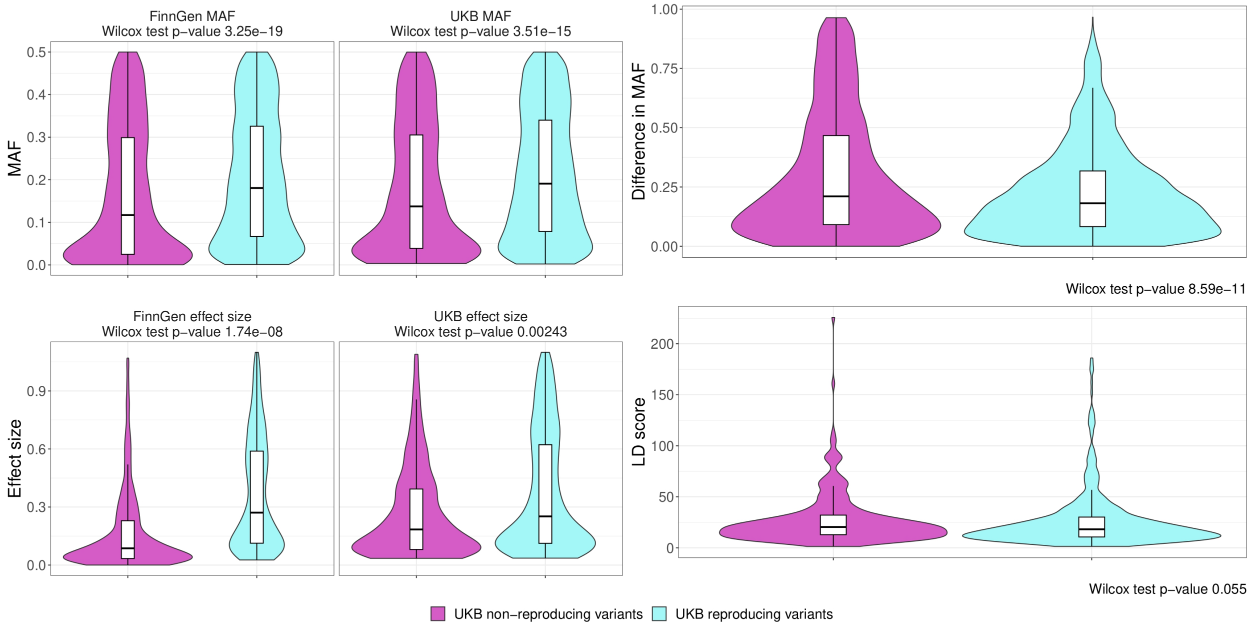

Supplementary figure S5. Comparison of variant-level properties between UK Biobank-sourced index SNPs at loci with reproducible (blue) and non-reproducible (purple) associations. Plots show (a) minor allele frequency (MAF) (left - FinnGen (FG), right - UK Biobank (UKB)); (b) scaled difference in MAF between UKB and FG; (c) variant effect sizes (left - FG, right - UKB); and (d) variant LD score

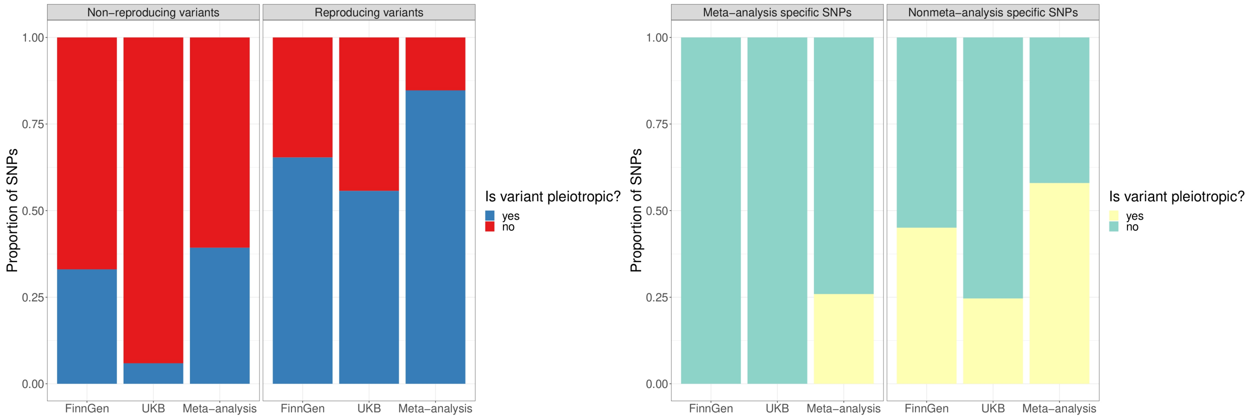

Supplementary figure S6. Barplots showing the proportion of pleiotropic and non-pleiotropic variants among different groups of variants. On the left: reproducible variants vs. non-reproducible variants; on the right: meta-specific associations vs all other associations.
